# Draft Genome Sequence of *Aeromonas hydrophila* TN97-08

**DOI:** 10.1128/genomeA.00436-16

**Published:** 2016-05-26

**Authors:** Hasan C. Tekedar, Salih Kumru, Attila Karsi, Geoffrey C. Waldbieser, Tad Sonstegard, Steven G. Schroeder, Mark R. Liles, Matt J. Griffin, Mark L. Lawrence

**Affiliations:** a College of Veterinary Medicine, Mississippi State University, Mississippi State, Mississippi, USA; b Warmwater Aquaculture Research Unit, Agriculture Research Service, United States Department of Agriculture, Stoneville, Mississippi, USA; c Bovine Functional Genomics Laboratory, Agricultural Research Service, United States Department of Agriculture, Beltsville, Maryland, USA; d Department of Biological Sciences, Auburn University, Auburn, Alabama, USA

## Abstract

*Aeromonas hydrophila* is an opportunistic pathogen residing in freshwater environments that causes infection in fish and mammals. Here, we report the draft genome sequence of *A. hydrophila* strain TN97-08 isolated from a diseased bluegill (*Lepomis macrochirus*) in 1997.

## GENOME ANNOUNCEMENT

*Aeromonas hydrophila* is an opportunistic Gram-negative species causing disease in fish and mammals. The genus *Aeromonas* affects a variety of aquatic organisms and lives in diverse aquatic ecosystems (1). There are 45 *A. hydrophila* genomes currently available in GenBank. Our research group released the genomes of strains ML09-119 and AL06-06 (accession numbers CP005966 and CP010947). Strain ML09-119 was isolated from an outbreak of bacterial septicemia in a commercial channel catfish pond in the southeastern United States (2). Strain AL06-06 was isolated from a diseased goldfish in 2006 (3). Here, we report the draft genome sequence of strain TN97-08, which was isolated in 1997 from diseased bluegill (*Lepomis macrochirus*).

The *A. hydrophila* TN97-08 genome was sequenced using an Illumina Genome Analyzer IIx (9,280,112 reads with 243× coverage). Adaptor trimming, contig creation, and quality control of sequence reads were conducted using CLC Workbench 6.5.1 (CLC bio) and Sequencher 5.3 (Gene Codes Corporation). *De novo* assembly was performed by CLC Workbench 6.5.1. To reduce contig numbers, some of the scaffolded gaps were closed by Sanger sequencing of PCR amplicons. Additionally, some of the unscaffolded gaps were closed by single-primer PCR (4).

Two different methods were used to annotate the draft genome: RAST (5) and the NCBI Prokaryotic Genome Automatic Annotation Pipeline (PGAAP) (6). The draft genome of *A. hydrophila* TN97-08 is composed of 16 contigs and 5,087,310 bp. It contains 4,602 predicted genes, of which 4,445 are protein-coding genes.

The annotated *A. hydrophila* TN97-08 genome was compared against the genomes of *A. hydrophila* ML09-119 and AL06-06. The results indicated that *A. hydrophila* TN97-08 has 100 and 74 unique elements compared to strains ML09-119 and AL06-06, respectively. One of the unique features identified is a type VI secretion system, which is considered a virulence factor involved in the translocation of potential effector proteins into the host (7). Other unique elements include multidrug resistance efflux pumps, cobalt zinc cadmium resistance, stress response proteins, phage and prophage elements, and toxin-antitoxin replicon stabilization system elements. In contrast to the AL06-06 genome, TN97-08 does not carry any plasmids. The estimated average nucleotide identity (http://enve-omics.ce.gatech.edu/ani/) between the strain TN97-08 genome and the strain ML09-199 genome was 96.61%, and it was 96.72% between TN97-08 and AL06-06 (8).

### Nucleotide sequence accession numbers.

The draft genome sequence of *A. hydrophila* TN97-08 was deposited in GenBank under accession no. LNUR00000000, version LNUR00000000.1, genomic ID 982545319.
